# Molecular Determination of *Toxocara* spp. Eggs Isolated from Public Parks and Playgrounds in Zahedan, Southeast Iran

**DOI:** 10.1155/2024/2132696

**Published:** 2024-05-27

**Authors:** Siavash Liravizadeh, Samaneh Abdolahi Khabisi, Alireza Salimi Khorashad, Hadi Mirahmadi

**Affiliations:** Department of Parasitology and Mycology Faculty of Medicine Zahedan University of Medical Sciences, Zahedan, Iran

## Abstract

**Background:**

Human toxocariasis (HT) is a zoonotic disease with a global expansion. Contaminated soil with *Toxocara* spp. eggs is the main source of human infection, which may lead to severe complications depending on the organs invaded by migrating larvae.

**Aim:**

This study is aimed at eliciting the prevalence of *Toxocara* spp. eggs in public parks in Zahedan, southeast Iran, and providing new insight into the soil contamination rate in this area using microscopic and molecular methods.

**Methods:**

Based on five municipal districts, 240 soil samples were collected from public parks and playgrounds in Zahedan. The modified Sheather's flotation technique was employed to isolate *Toxocara* spp. eggs from the soil, followed by microscopic assessment and molecular evaluation of internal transcribed spacer 1 and 2 ribosomal deoxyribonucleic acid (ITS1 and 2 rDNA) using nested polymerase chain reaction (nested PCR) to identify the presence of *Toxocara* spp. eggs. The Sanger sequence was used to differentiate the *Toxocara* species. Subsequently, all the sequenced data were blasted and compared with other sequences available in the GenBank.

**Results:**

Out of 240 soil samples collected, 7 (2.9%) samples were identified to contain *Toxocara* spp. eggs using Sheather's flotation and microscopic techniques. Meanwhile, 19 (7.9%) samples were positive using nested PCR. According to the Sanger sequencing analysis findings, all positive samples were contaminated with *Toxocara cati*.

**Conclusion:**

As evidenced by the obtained results, only *T. cati* species were detected in public parks and playgrounds in Zahedan; therefore, control and prevention programs against this species should be considered in human and animal communities.

## 1. Introduction

*Toxocara* spp. are mammalian roundworm parasites that reside in the small intestines of canines and cats across the globe. As definitive hosts, *T. cati* and *T. canis* are the two most prevalent zoonotic species associated with human toxocariasis (HT) infection [1]. The definitive host expels unembryonated eggs into the soil through the feces, while the accidental swallowing of embryonated eggs existing in the contaminated soil and unwashed plant-based foods (vegetables and raw paratenic flesh), as well as chicken and hare, might lead to human infection [2].

Depending on parasite load and subsequent immigration spots of larvae, HT leads to a broad spectrum of clinical syndromes and complications, including visceral larva migrans (VLM), ocular larva migrans (OLM), covert toxocariasis, neurotoxocariasis, cardiac disorder, and asthma [3–6]. Based on epidemiologic studies, the estimated global seroprevalence rate was 19.0%, with maximum and minimum prevalence rates of 37.7% and 8.2% in African and Eastern Mediterranean regions, respectively. Based on the results of 19-year research, this seroprevalence was 9.3% in Iran [7, 8].

As far as soil contamination is concerned, public places, such as parks, playgrounds, and sandboxes, which are common for pet walking and stray dogs and cats, are known as the most likely sites to increase the chance of infection posed by exposure to *Toxocara* spp. eggs, especially for children, due to their increased likelihood of ingesting soil and closer interaction with animals [9]. Therefore, soil is recognized as a potential infection-transmitting source for humans, and it needs to be closely observed. The recognition of *Toxocara* spp. eggs in soil by microscopic and molecular methods is essential for the development of preventative and control measures for human and animal communities. On the other hand, *Toxocara* spp. eggs are difficult to differentiate from eggs of other species in soil via microscopic methods due to their size and morphological resemblance; therefore, other proper techniques must be considered. Molecular techniques, as well as polymerase chain reaction- (PCR-) loop-mediated isothermal amplification (LAMP), nested PCR, PCR-restriction fragment length polymorphism (RFLP), and PCR sequencing, are highly recommended as reliable approaches for the detection of *Toxocara* spp. eggs [10].

In addition, the pooled global prevalence of soil contamination was reported as 21% in Iran, but the precise contamination status of soil in Zahedan is unknown. In light of the aforementioned issues, the present study is aimed at eliciting the prevalence and distribution of *Toxocara* spp. eggs in public parks in Zahedan, southeast Iran, in an attempt to provide new insight into the rate of soil contamination in this area.

## 2. Materials and Methods

### 2.1. Sample Collection

**Figure 1:**
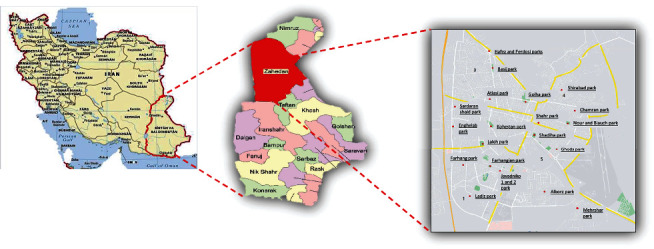
The map of the studied parks from five municipal districts of Zahedan for soil sampling.

Zahedan, the capital of Sistan and Baluchestan Province, is located in the southeast of Iran. This cross-sectional study was carried out from March to May and August to October 2022 (code of ethics: IR.ZAUMS.REC.1401.201). The sample size was calculated according to a study conducted in Yazd (central Iran), which resembles Zahedan in temperature and climate [11]. A total of 20 public parks, playgrounds, and recreational sites (with inclusion criteria bigger than 2500 m^2^) were selected from five municipal districts of Zahedan (Figure 1). Considering the accuracy rate of 25% (0.25 is correct) and design effect of 1.2%, the number of required soil samples was estimated to be 240 based on the sample size calculation formula. A total of 12 soil samples were gathered from the top 3-5 cm of soil in each park using a shovel to ensure an even sampling of the entire area. Each sample (300 g) was placed in a plastic container and labeled before being transported to the laboratory.

### 2.2. Sheather's Flotation Technique

In the laboratory, the collected soil samples were dried at room temperature for 24 h and passed through a100 *μ*m mesh sieve. A modified Sheather's flotation technique [12] was used to sort out *Toxocara* spp. eggs from soil samples. According to this technique, 10 g of soil samples was thoroughly mixed with 40 ml of Tween 20 (0.5%) and centrifuged for 10 min at 1500 rpm in a 50 ml conical centrifuge tube, and the supernatant was then decanted. The same procedure was performed with distal water, and the supernatant was decanted as well. Following that, 40 ml of a saturated sugar solution (specific gravity: 1.27) was added to the sediment, mixed, and centrifuged for 10 min at 1500 rpm. The volume was increased to 45 ml by adding extra sugar solution and then centrifuged for 5 min at 800 rpm. Subsequently, the top 10 ml of supernatant was pipetted and transferred into a clean conical centrifuge tube. The 10 ml transferred supernatants were washed (centrifuged for 5 min at 800 rpm) once with 40 ml of distal water again. After decanting all supernatants, the sediment of each sample was stored at -20°C for further microscopic and molecular evaluation. In terms of microscopic assessment, the observation and identification of *Toxocara* spp. eggs were performed at magnifications of 100x and 400x based on morphological futures.

### 2.3. DNA Extraction and Nested Polymerase Chain Reaction

All flotation fluids (obtained from the Sheather's flotation technique) were frozen and thawed three times using liquid nitrogen for 3 minutes and boiling water for 5 minutes, followed by overnight proteinase K digestion. Following that, according to the manufacturer's instructions, genomic DNA was extracted using the Tissue Genomic DNA Extraction Mini Kit (Yekta Tajhiz Azma, Tehran, Iran, Lot. No. CB311121505). Internal transcribed spacers 1 and 2 (ITS1 and ITS2), as well as the ribosomal DNA region, were amplified using nested PCR. In the first step of nested PCR, the forward primer (NC5: 5′-GTAGGTGAACCTGCGGAAGGATCATT-3′) and reverse primer (NC2: 5′-TTAGTTTCTTTTCCTCCGCT-3′) were used to amplify the ITS region [13]. The first PCR were accomplished in a final volume of 25 *μ*l. The reaction mixture was prepared as follows: 12.5 *μ*l of PCR Master Mix (2 Master Mix RED; Sinaclon, Iran, Cat. No. MM2062), 0.7 *μ*l (12.5 pmol) of each primer, 5 *μ*l of DNA template, and 6.1 *μ*l double distal water. The temperature profile was a single cycle of 95°C for 6 min as primary denaturation, followed by 35 cycles of 94°C for 45 sec (denaturation), 60°C for 1 min (annealing), 72°C for 1 min (extension), and a final extension of 72°C for 6 min.

The secondary nested PCR were performed using forward primer (FM1: 5′-TTGAGGGGAAATGGGTGAC-3′) and reverse primer (FM2: 5′-TGCTGGAGGCCATATCGT-3′) in a 25 *μ*l reaction volume [14]. A volume of 5 *μ*l of the first-step product was used as the template for the second step of nested PCR. As an initial step, preheating at 94°C for 6 min was performed, followed by 35 cycles of denaturation at 94°C for 45 sec, annealing at 60°C for 60 sec, and extension at 72°C for 1 min, with a final extension step of 6 min at 72°C. The PCR products were electrophoresed on a 1.5% agarose gel and stained with safe stain. The gel was placed in the gel doc chamber for UV exposure.

### 2.4. Sequencing and Alignment

The PCR products were purified using the DNA gel extraction (FAVORGEN, Cat. No. FAGCK001.1) according to the manufacturer's instructions and sent to Microsynth Biotechnology Company (Swiss) for the Sanger sequencing. All the sequenced data were blasted and compared with other available sequences in the GenBank. Eight sequenced samples were successfully submitted to the gene bank database. Isolates from this study were aligned with *Toxocara cati* isolates from Shiraz, Iran, using BioEdit software (version 7.2.5).

## 3. Results

### 3.1. Microscopic Assessment

**Figure 2:**
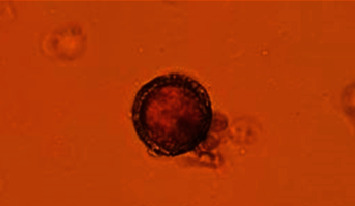
*Toxocara* spp. egg was isolated from soil using Sheather's flotation technique.

Out of 240 samples collected from 20 local parks, playgrounds, or recreational sites across Zahedan, *Toxocara* spp. eggs were detected in 7 (2.9%) samples.  Figure 2 shows *Toxocara* spp. egg using Sheather's flotation and microscopic techniques from soil sample.

Table 1 shows the frequency of soil samples contaminated with *Toxocara* spp. from Zahedan public park using both Sheather's flotation and nested PCR techniques.

### 3.2. Nested Polymerase Chain Reaction and Sequencing

**Figure 3:**
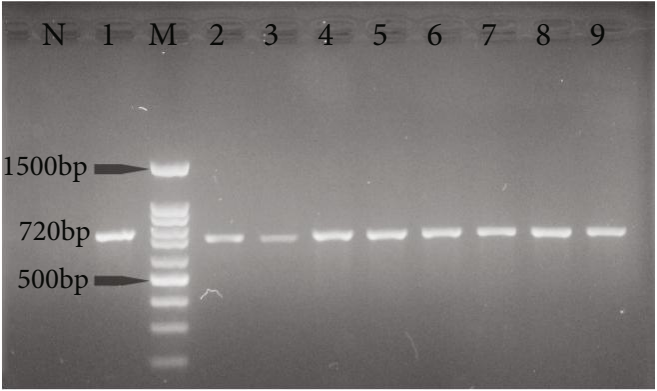
Agarose gel electrophoresis of the second step of nested PCR products. M: 100 bp DNA marker; N: negative control; lane 1: positive control; lanes 2–9: *Toxocara* spp. infected soil samples (yielded amplicons of approximately 700 bp).

All soil samples were examined using the nested PCR technique, which targeted a specific area of ribosomal DNA known as the ITS1 and ITS2 regions. The number of 19 (7.9%) samples yielded amplicons of approximately 700 bp using nested PCR (Figure 3).

**Figure 4:**
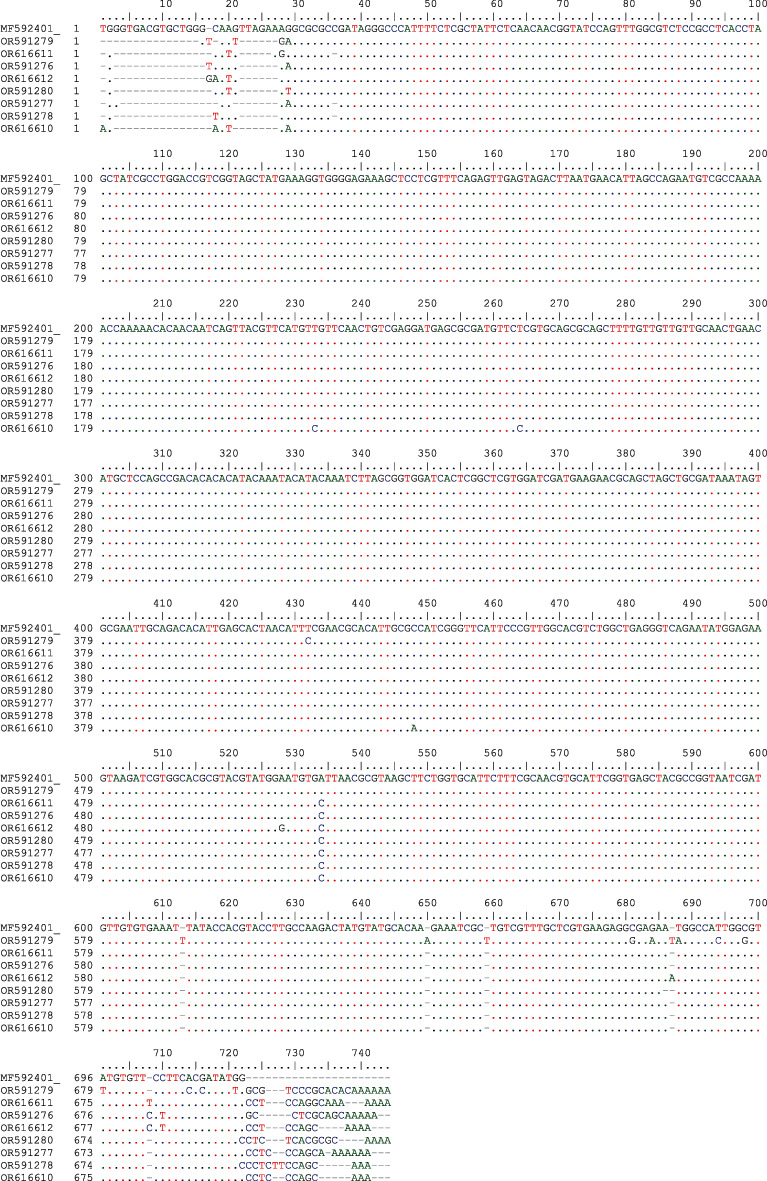
Alignment of sequences of the ITS1 and ITS2 genes of *T. cati* (OR591276, OR591277, OR591278, OR591279, OR591280, OR616610, OR616611, and OR616612) isolated from soil samples at the Zahedan, Iran, with *T. cati* isolated (MF592401) from Shiraz, Iran.

All the blasted sequences were *T. cati* species. Eight accession numbers, including OR591276, OR591277, OR591278, OR591279, OR591280, OR616610, OR616611, and OR616612, were submitted to the GenBank database. All accession numbers demonstrated more than 97% identity with the available sequences of *T. cati*, including KY003092.1 reported from Guangzhou, China; JX536259 reported from Tehran, Iran; KJ777179.1 reported from Dring, India; and MF592401 reported from Shiraz, Iran (Figure 4).

## 4. Discussion

Contaminated soil with *Toxocara* spp. eggs is the main source of human infection, which may lead to severe complications depending on the organs invaded by migrating larvae. The rate of soil contamination with *Toxocara* spp. eggs is affected by sample collection, concentration method, soil type, cultural level, religious, behavioral patterns, temperature, humidity, and public health conditions [15, 16]. Several studies have been conducted around the world and in Iran to evaluate the seroprevalence of HT. According to these studies, the prevalence rates of HT by the serological method have been estimated to be 19% worldwide and 9.3% in Iran and the prevalence rate of 1.3% in Zahedan [7, 8, 17].

Recent studies indicated that the seroprevalence of HT in Iran, particularly in Zahedan, is comparatively lower than the global average. In many Islamic countries, such as Iran, interaction with dogs and cats is often restricted due to religious norms. Furthermore, Iran is classified as an area with high temperatures and low humidity, which reduces the viability and survival of *Toxocara* spp. eggs in the soil. In the current study, *Toxocara* spp. eggs were detected in 2.9% of the soil of parks and playgrounds in Zahedan using a Sheather (saturated sucrose) flotation assay.

The results of several studies on the rate of soil contamination with *Toxocara* spp. eggs in Iran are consistent with those of the current research. A total of 4%, 5%, 7%, and 3.7% of soil samples in Shiraz, Chaharmahal and Bakhtiari, Ardabil, and Sari in the north of Iran have been reported to be positive for *Toxocara* spp. eggs, respectively, using the saturated sucrose flotation method [14, 18–20]. On the contrary, the rates of soil contamination with *Toxocara* spp. eggs in Hamedan, Abadan, Kermanshah, Iraq, the Philippines, England, Brazil, Lisbon, and New York City have been determined at 29.2%, 11.8%, 18%, 22.2%, 77%, 86.6%, 53%, 38.5%, and 18.3%, respectively [1, 21–28].

Differences in climate, culture, and religious beliefs about pet ownership can affect the rate of soil contamination. Temperature and humidity play a prominent role in the rate of soil contamination with *Toxocara* spp. eggs. The environmental conditions affect the survival of *Toxocara* spp. eggs.

The studies in Tehran and Lisbon have demonstrated a rise in eggs inside the soil during spring and autumn [28, 29]. Therefore, in the present study, samples were collected during the spring and autumn. In the current study, there were several limitations, such as dry weather and low rainfall in Zahedan, which were expected to reduce the number of eggs in the soil. It should be mentioned that we overcame this limitation by sampling in the best season and using sensitive molecular methods to detect *Toxocara* spp. eggs.

It is worth noting that although spring and autumn are the best seasons in Zahedan in terms of temperature, humidity, and rainfall, the rates of soil contamination with *Toxocara* spp. eggs in Zahedan parks during these specific seasons were low. Areas, such as Zahedan, with low humidity, limited rainfall (compared to average global rainfall), and inhospitable climates, even in these two seasons, exhibited reduced soil contamination, pointing to the effect of climate and weather on the spread of *Toxocara* spp. eggs. Moreover, low rates of soil contamination with *Toxocara* spp. eggs have been reported in other regions with a climate similar to Zahedan.

Although the majority of investigations on *Toxocara* spp. eggs in contaminated soil have focused on microscopic examination as a diagnostic technique, the outcomes of this approach have yielded limited reliability. Due to the fact that *T. cati* and *T. canis* eggs are nearly identical in size and shape, the identification of *Toxocara* spp. is difficult using the microscopic method. Molecular methods, such as PCR-RFLP, PCR sequencing, and PCR using species-specific primers, are appropriate for species identification as a reliable technique. Furthermore, species determination is necessary for adopting control and prevention programs against toxocariasis in humans and animals.

In the present study, *Toxocara* spp. eggs were detected in 7.9% of the soil from parks and playgrounds in Zahedan using nested PCR. In studies conducted in Shiraz, Ahvaz, Tabriz, Poznan, and Lisbon, microscopic and molecular methods were used; concordantly, all surveys represent additional accuracy of the molecular method [14, 28, 30–32].

In the current study, we were able to report a higher amount of parasites in the soil due to the higher sensitivity of the molecular technique. In this study, all positive samples were *T. cati* using the Sanger sequencing method. This can be ascribed to the fact that the majority of parks are situated in the center of the city, where cats are more prevalent in the urban setting compared to dogs. In contrast, dogs are predominantly observed on the outskirts of the city. Furthermore, the presence of religious constraints regarding dog ownership had a significant impact on this issue.

In Portugal, 53% of collected soil samples were contaminated with *T. cati* using a sequencing assay, and no other species were detected. In Shiraz and Lisbon, the rates of contaminated soil by the molecular method were 16% and 53%, respectively, and the most commonly observed species was *T. cati* using PCR-RFLP [14, 28]. Consistent with our study, in Khouzestan, soil contamination with *T. cati* (28%) was more prevalent than with *T. canis* (5.7%). The rates of soil contamination in northwest Iran with *T. canis*, *T. cati*, and mixed infection were reported to be 15.5%, 27.2%, and 12.2%, respectively, using the LAMP assay [30, 31].

In the current study, the Sanger sequencing technique enabled us to determine the species and genetic sequence of *Toxocara* spp. in Zahedan for the first time. Inconsistent with the current study, in Isfahan, the overall rate of contaminated soil was 21.69%, with 12.39% attributed to *T. canis* and 8.73% to *T. cati* using the conventional PCR method with two pairs of specific primers for each species [33]. Furthermore, considering the survey performed in England, *T. canis* was detected in all positive samples using the Sanger sequencing technique [26]. This study's strengths include using a sensitive molecular method (nested PCR) and sequencing to determine the soil contamination rate and *Toxocara* species. Also, one of the study's weak points is the need for more information about the infection rate of dogs and cats with *Toxocara* spp. in Zahedan. Therefore, to overcome this problem, *Toxocara* spp. infection in dogs and cats should be investigated.

## 5. Conclusion

In the current study, a higher rate of soil contamination with *Toxocara* spp. was observed using the nested PCR method compared to the flotation concentration assay. Therefore, the nested PCR method is more sensitive compared to the flotation method. In addition, only *T. cati* species were detected in public parks and playgrounds in Zahedan. Therefore, control and prevention programs should be planned against this species in human and animal communities in Zahedan. It is noteworthy that the infection rate of cats and dogs in Zahedan is unknown. Feature molecular and microscopic assays should be conducted on animal stool exams to clarify the *Toxocara* spp. infection rate in Zahedan.

## Figures and Tables

**Table 1:** Frequency of soil samples contaminated with *Toxocara* spp. from Zahedan public parks using both of Sheather's flotation and nested PCR techniques.

Public parks	The number of soil samples examined in each park	Sheather's flotation method		Nested PCR method	
		Positive	%	Positive	%
Ladiz	12	0	0	0	0
Farhang	12	0	0	1	8.3
Farhangian	12	0	0	0	0
Shahid Javad Nikoo	12	0	0	1	8.3
Laleh	12	2	16.6	2	16.6
Kohestan Park	12	1	8.3	1	8.3
Sardaran Shahid	12	0	0	0	0
Enghelab	12	1	8.3	3	12
Atlasi	12	0	0	2	16.6
Golha	12	1	8.3	2	16.6
Basij	12	1	8.3	2	16.6
Yadegar Emam	12	0	0	1	8.3
Shir Abad	12	0	0	0	0
Chamran	12	0	0	0	0
Park Shahr	12	0	0	0	0
Noorbalooch	12	1	8.3	2	16.6
Shadi	12	0	0	1	8.3
Ghods	12	0	0	0	0
Zibashahr	12	0	0	1	8.3
Mehrshahr	12	0	0	0	0
Total	240	7	2.9	19	7.9

## Data Availability

The data that support the findings of this study are available on request from the corresponding author.

## References

[1] Tyungu D. L., McCormick D., Lau C. L. (2020). Toxocara species environmental contamination of public spaces in New York City. *PLoS Neglected Tropical Diseases*.

[2] Strube C., Heuer L., Janecek E. (2013). Toxocara spp. infections in paratenic hosts. *Veterinary Parasitology*.

[3] Despommier D. (2003). Toxocariasis: clinical aspects, epidemiology, medical ecology, and molecular aspects. *Clinical Microbiology Reviews*.

[4] Li S., Sun L., Liu C. (2021). Clinical features of ocular toxocariasis: a comparison between ultra-wide-field and conventional camera imaging. *Eye (London, England)*.

[5] Auer H., Walochnik J. (2020). Toxocariasis and the clinical spectrum. *Advanced Parasitology*.

[6] Mazur-Melewska K., Mania A., Sluzewski W., Figlerowicz M. (2020). Clinical pathology of larval toxocariasis. *Advanced Parasitology*.

[7] Rostami A., Riahi S. M., Holland C. V. (2019). Seroprevalence estimates for toxocariasis in people worldwide: a systematic review and meta-analysis. *PLoS Neglected Tropical Diseases*.

[8] Eslahi A. V., Badri M., Khorshidi A. (2020). Prevalence of Toxocara and Toxascaris infection among human and animals in Iran with meta-analysis approach. *BMC Infectious Diseases*.

[9] Raissi V., Masoumi M. T., Ibrahim A. (2021). Spatial analysis of Toxocara spp. eggs in soil as a potential for serious human infection. *Infectious Diseases*.

[10] Chen J., Zhou D. H., Nisbet A. J. (2012). Advances in molecular identification, taxonomy, genetic variation and diagnosis of Toxocara spp. *Infection, Genetics and Evolution*.

[11] Mohaghegh M.-A., Norouzi R., Siyadatpanah A., Mirzaei F., Fatahi Bafghi A., Mirbadie S.-R. (2021). Soil contamination with eggs of Toxocara spp. in Yazd, Central Iran. *Medical Laboratory Journal*.

[12] Zibaei M., Bahadory S., Sadjjadi S., Heidari A., Hosseini H. (2019). Designing the vertical sieve screening in order for recovery of Toxocara spp. eggs from soil samples. *Journal of Veterinary Research*.

[13] Li M. W., Lin R. Q., Chen H. H., Sani R. A., Song H. Q., Zhu X. Q. (2007). PCR tools for the verification of the specific identity of ascaridoid nematodes from dogs and cats. *Molecular and Cellular Probes*.

[14] Choobineh M., Mikaeili F., Sadjjadi S. M., Ebrahimi S., Iranmanesh S. (2019). Molecular characterization of Toxocara spp. eggs isolated from public parks and playgrounds in Shiraz, Iran. *Journal of Helminthology*.

[15] Fakhri Y., Gasser R. B., Rostami A. (2018). Toxocara eggs in public places worldwide - a systematic review and meta-analysis. *Environmental Pollution*.

[16] Macpherson C. N. L. (2013). The epidemiology and public health importance of toxocariasis: a zoonosis of global importance. *International Journal for Parasitology*.

[17] Salimi Khorashad A., Shahraki M., Rahmati Balaghaleh M. (2021). Seroprevalence of Toxocara spp. in children (3-13 years old) in Zahedan, southeast of Iran. *Journal of Parasitic Diseases*.

[18] Shirvani G., Abdizadeh R., Manouchehri Naeini K., Mortezaei S., Khaksar M. (2019). The study of soil contamination by Toxocara spp. eggs in different areas of Chaharmahal and Bakhtiari Province, Southwest Iran. *International Journal of Epidemiologic Research*.

[19] Pezeshki A., Haniloo A., Alejafar A., Mohammadi-Ghalehbin B. (2017). Detection of Toxocara spp. eggs in the soil of public places in and around of Ardabil City, northwestern Iran. *Iranian Journal of Parasitology*.

[20] Hezarjaribi H. Z., Daryani A., Kelarijani N. A. (2018). Parasitic contamination of surface and deep soil in different areas of Sari in north of Iran. *Journal of Coastal Life Medicine*.

[21] Sazmand A., Torkaman S., Namayeshi S., Faraji S., Zeinali M., Zibaei M. (2020). Prevalence of Toxocara species eggs in the soil of public parks in Hamedan City, Western Iran. *Avicenna Journal of Clinical Microbiology and Infection*.

[22] Maraghi S., Mazhab Jafari K., Sadjjadi S. M., Latifi S. M., Zibaei M. (2014). Study on the contamination of Abadan public parks soil with Toxocara spp. eggs. *Journal of Environmental Health Science & Engineering*.

[23] Ghashghaei O., Khedri J., Jahangiri-Nasr F., Hashemi S., Nourollahi Fard S. R. (2016). Contamination of soil samples of public parks with Toxocara spp. eggs in Kermanshah, Iran. *İstanbul Üniversitesi Veteriner Fakültesi Dergisi*.

[24] Taher H. (2017). Soil contamination with intestinal parasites eggs in public parks and playgrounds in Kirkuk city. *Tikrit Journal of Pure Science*.

[25] Paller V. G., de Chavez E. R. (2014). *Toxocara* (Nematoda: Ascaridida) and other soil-transmitted helminth eggs contaminating soils in selected urban and rural areas in the Philippines. *The Scientific World Journal*.

[26] Airs P. M., Brown C., Gardiner E., Maciag L., Adams J. P., Morgan E. R. (2023). WormWatch: park soil surveillance reveals extensive Toxocara contamination across the UK and Ireland. *Veterinary Record*.

[27] Delai R. R., Freitas A. R., Kmetiuk L. B. (2021). One health approach on human seroprevalence of anti-Toxocara antibodies, Toxocara spp. eggs in dogs and sand samples between seashore mainland and island areas of southern Brazil. *One Health*.

[28] Otero D., Alho A. M., Nijsse R., Roelfsema J., Overgaauw P., Madeira de Carvalho L. (2018). Environmental contamination with Toxocara spp. eggs in public parks and playground sandpits of Greater Lisbon, Portugal. *Portugal Journal of Infection and Public Health*.

[29] Raissi V., Saber V., zibaei M. (2020). Comparison of the prevalence of Toxocara spp. eggs in public parks soils in different seasons, from 2017 to 2018, Tehran Province, Iran. *Clinical Epidemiology and Global Health*.

[30] Khademvatan S., Abdizadeh R., Tavalla M. (2014). Molecular characterization of Toxocara spp. from soil of public areas in Ahvaz southwestern Iran. *Acta Tropica*.

[31] Ozlati M., Spotin A., Shahbazi A. (2016). Genetic variability and discrimination of low doses of Toxocara spp. from public areas soil inferred by loop-mediated isothermal amplification assay as a field-friendly molecular tool. *Veterinary World*.

[32] Mizgajska-Wiktor H., Jarosz W., Fogt-Wyrwas R., Drzewiecka A. (2017). Distribution and dynamics of soil contamination with Toxocara canis and Toxocara cati eggs in Poland and prevention measures proposed after 20 years of study. *Veterinary Parasitology*.

[33] Pourshahbazi G., Khanahmad H., Khadivi R. (2022). Environmental contamination of different areas of Isfahan Province of Iran with Toxocara spp. *Eggs using Molecular Methods. Advanced Biomedical Research*.

